# Oral Habits in Childhood and Occlusal Pathologies: A Cohort Study

**DOI:** 10.3390/clinpract14030057

**Published:** 2024-04-24

**Authors:** Mario Santos Barrera, David Ribas-Perez, Carolina Caleza Jimenez, Olga Cortes Lillo, Asunción Mendoza-Mendoza

**Affiliations:** Department of Stomatology, University of Seville, 41004 Seville, Spain

**Keywords:** oral habits, functional habits, sucking habits, malocclusions

## Abstract

Purpose: To analyse the relationship between the different habits that occur in childhood and the different malocclusions in the three planes of space. Material and methods: A clinical examination of 106 children between 5 and 12 years of age was carried out and a survey validated by professors of the Faculty of Dentistry of the University of Seville was made for the parents in order to identify the habits and relate them to the possible malocclusions detected in the child’s mouth. Results: 72.64% of the sample presented a malocclusion in at least one of the three planes of space, with a similar distribution. When correlating the variables, statistically significant relationships were observed in the vertical plane with atypical swallowing (*p* = 0 < 0.05; V > 0.3) and lip sucking (*p* = 0 < 0.05; V > 0.3) and in the horizontal plane with oral breathing (*p* = 0 < 0.05; V > 0.3), atypical swallowing (*p* = 0 < 0.05; V < 0.3) and digital sucking (*p* = 0 < 0.05; V < 0.3). Conclusions: It has been observed that the prevalence and prolongation of habits in childhood is increasing, so it is essential to detect pernicious habits at an early age to prevent the establishment of malocclusions and to favour the correct craniofacial growth of the child.

## 1. Introduction

Malocclusion is a misalignment of the upper and lower teeth, where an adequate gear of bone structures between the maxilla and jaw is not observed, resulting in the lack of an ideal function of the masticatory apparatus. This condition is considered a public health problem because it causes psychological problems and affects quality of life. Malocclusions can be classified according to the three planes of space: vertical, transverse and sagittal [1].

Vertical malocclusions are classified as open bite and deep bite. The open bite is a condition which one or more teeth cannot contact their antagonists and is related to the mouth breathing habit when the airway obstruction is not resolved. In contrast, the deep bite has an increased vertical overbite and boys have more overbite than girls. On the other hand, transverse malocclusions are disorders where the upper and lower posterior teeth do not occlude properly and are associated with prolonged sucking habits. They are classified as unilateral posterior, bilateral posterior crossbite and scissor bite (Brodie bite). Moreover, sagittal malocclusion is classified according to Angle’s classification into Class I, Class II and Class III malocclusions. Class I is where the mesial cusp of the upper first molar occludes between the mesial and median cusps of the first lower molar; Class II is when the permanent lower molar occludes distally from its normal position. Finally, Class III manifests at the inversion of dental relationships, by the first permanent lower molar that is mesially occluded with respect to its normal position [2].

With regard the aetiology of malocclusions, deleterious habits can interfere with the dental structure and may in part cause an imbalance in muscle strength and changes in the normal functional aesthetics of the entire stomatognathic apparatus [3]. Therefore, physiological or functional habits are those that are related to a rupture of muscular balance that generates a functional alteration that affects the nasal, buccal, and pharyngeal spaces. Among them are oral breathing and atypical swallowing [4].

It is important to highlight oral breathing. It has a complex aetiology and may be due to genetic factors, nasal obstruction, respiratory allergies, or a poor sleeping position. Although some authors believe that the change in the normal pattern of dentoskeletal growth is due to genetic and environmental factors, most think instead that the obstruction of the upper airways, resulting in mouth breathing, changes the pattern of craniofacial growth with typical facial features and dentition [5]. At the dental level, the interposition of the lower lip causes the retroposition and verticalisation of the lower incisors and an increased protrusion of the upper incisors. At the skeletal level, we observe the appearance of a uni- or bilateral crossbite, an anterior bite, and an Angle Class II malocclusion [6]. These correlations require a close collaboration between orthodontics and other professionals such as paediatricians, otorhinolaryngologists, and posturologists.

Another habit is atypical swallowing which is considered persistent because of a delay in maturation acting as an infantile swallow. It is associated with an anterior open bite, speech disturbance and the obvious protrusion of the upper incisors [7]. As for dyslalia, it is any alteration that affects speech development, hindering and distorting the pronunciation of different sounds. Factors that influence this disorder are the presence and position of the teeth (especially the anterior teeth), the mobility of the lips, cheeks, the soft palate, tongue, and jaw [4,8].

Finally, sucking habits consist of external modifiers that play an important role in craniofacial development and growth. They are classified as nutritive (breastfeeding and bottle feeding) and non-nutritive (pacifier, digital sucking, and lip sucking) [8]. Breastfeeding is a habit that brings general, immunological, nutritional and oral benefits to the child. In fact, the World Health Organization (WHO) recommends exclusive breastfeeding for the first 6 months to 2 years [9]. It has been shown to reduce malocclusions and improve mastication quality due to the better development of the masseter, temporalis, and pterygoid muscles [10,11,12]. The alternative to breastfeeding in cases where this is not possible is bottle feeding. During bottle feeding, the milk is expressed by compressing the teat, making a more passive movement to obtain the milk and causing less stimulation of the orofacial structures. Thus, the position of the tongue is lower than in breastfeeding, which means a weak stimulation of the tongue, jaw and muscles such as masseter and pterygoid [13]. Furthermore, since there is no synchronisation between swallowing and nasal breathing, many authors state that an oral breathing pathway is likely to be established [14]. With regard to pacifier use, its prolongation is common to cause some type of malocclusion [15,16]. The most frequent malocclusions due to prolonged use of pacifiers are an open anterior bite, a posterior bite (more significant when digital sucking is also present and due to nocturnal use), increased protrusion, distal molar relationship, and increased distance between canines with evidence of palatal compression [17]. When referring to digital sucking, it is important to know that it is considered a normal habit in the first two years. Subsequently, children discontinue this habit as they develop self-control skills [18]. People who suck for 6 h or more per day have been reported to have considerably more serious malocclusions, because the tongue adopts a forward posture, whose thrust next to the finger often leads to an asymmetric open anterior bite, which is worse on the side where thumb sucking occurs [14]. Furthermore, finger pressure causes retrognathia, the protrusion of the maxilla, the proinclination of the upper incisors, and the retroinclination of the lower incisors, further pronouncing the open bite [9]. The increased likelihood of developing an increased interincisor protrusion, a Class II canine relationship and a posterior crossbite is also common [8]. Finally, lip suction is often generated secondary to an atypical swallowing habit or digital suction. If the lip suctioned is the lower lip, the upper incisor protrusion will increase, while if the lip suctioned is the upper lip, the upper incisors will be retroinclined [18].

Due to the strong relationship between the presence of different habits with the appearance of malocclusions and the importance of early detection, we have carried out this study with the aim of analysing the prevalence of oral habits (functional or sucking) in childhood and identifying the different dentofacial alterations that can be triggered by each.

## 2. Materials and Methods

### 2.1. Data Collection

In this cohort study, data were collected from students at the Natalia Albanés School of Infant and Primary Education (Seville, Spain) by the same practitioner (M.S.B.) between November 2021 and December 2021.

Previously, we provided the parents/guardians with information on this research study with a questionnaire. This survey with 20 questions consisted of an anamnesis to find out the previous and current state of the child, as well as questions about the different habits. The initial survey was previously designed and validated by three professors from the Faculty of Dentistry of Dentistry of the University of Seville who modified the tool proposed by Alarcon et al. [19]. It received elevated levels in all the questions for coherence, clarity, and relevance. Once validated, the pilot phase was carried out by conducting a survey of 15 parents. There was no question that generated doubts when answering them.

Once their child had given permission to participate in the study, the identified cases that met the inclusion and exclusion criteria were assigned a research subject number (Table 1).

The children were examined intraorally and extraorally to assess the possible relationship between childhood habits and the presence of malocclusions. To carry out this methodology, a classroom was reserved exclusively for convenient examinations in natural light. Disposable examination mirrors were used to separate the lips and mucosa to inspect the molar class of the first permanent molars or the terminal plane of the E’s. The periodontal probe was only used to measure the millimetres of the predominant incisor protrusion. In addition, a photographic record was made with a Canon EOS 2000D SLR camera (Canon Europa N.V., Amstelveen, The Netherlands) with the aid of lip retractors on the intraoral photographs. A form was filled out for each of the children examined determining the type of dentition, and the existence of a vertical, horizontal and/or sagittal malocclusion.

We conducted the study in the Faculty of Dentistry of the University of Seville between November 2021 to May 2022, according to the Declaration of Helsinki and the Ethics Committee of the University of Seville (Code 0071-N-24). 

### 2.2. Statistical Analysis

Data were entered into an MS Excel spreadsheet to generate the study database, and errors or incomplete responses were checked. The SPSS version 25 statistical package was used to perform the descriptive analysis, calculating the frequencies for each variable. Contingency tables were generated to evaluate statistical associations between variables, based on the chi-square test. Considering the type of variables involved (mostly dichotomic qualitative variables), a descriptive study was carried out, with the calculation of the mean and standard deviation (SD) and percentages. Pearson’s chi-square test was used for hypothesis testing, considering a value of *p* < 0.05 as statistically significant. In addition, Cramer’s statistic was used to evaluate the strength of the statistical association between variables, where we defined an intense association defined by values of V > 0.3 and very intense for V > 0.5.

## 3. Results

### 3.1. Sample Description

**Figure 1:**
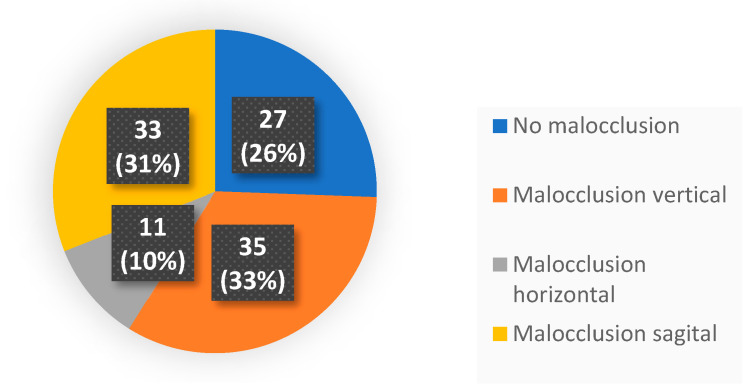
Pie diagram of the different malocclusions found in our study.

The final sample size was *n* = 106, where 52.8% represented the male sex and 47.2% the female sex. The mean age was 7.55 years with a deviation of 2.017. It should be noted that most of the sample presented some type of malocclusion in any of the three planes, occupying a large 72.64% of the study population, with a fairly similar distribution (Figure 1).

Data were collected using a standardised form that included patient age and sex, type of dentition, nutritive and non-nutritive sucking habits, and malocclusions in the vertical, horizontal, and sagittal planes (Table 2).

### 3.2. Variable Correlations

Once the sample was described, we related them to the types of malocclusions in the three planes of space.

**Figure 2:**
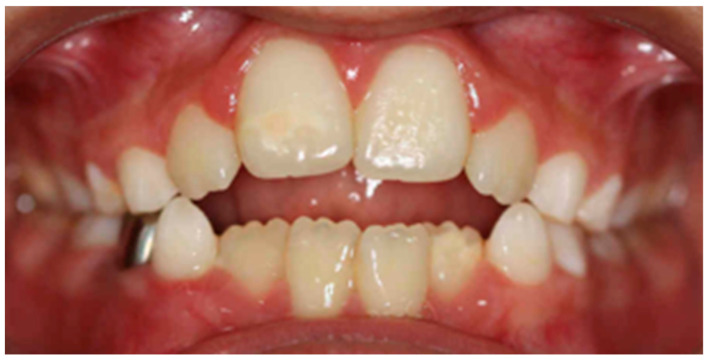
Intraoral photograph of an anterior open bite caused by immature deglutition.

In the vertical plane, it was determined that there is a statistically significant relationship between an immature swallowing habit and malocclusions in the vertical plane (*p* < 0.05), with an open bite being the most frequent, with a considerable strength of relationship between these variables (V > 0.3) (Figure 2). The same occurs with the lip sucking habit, with which there is a statistically significant relationship with vertical plane malocclusions (*p* < 0.05) and an intense relationship (V > 0.3) (Table 3). 

**Figure 3:**
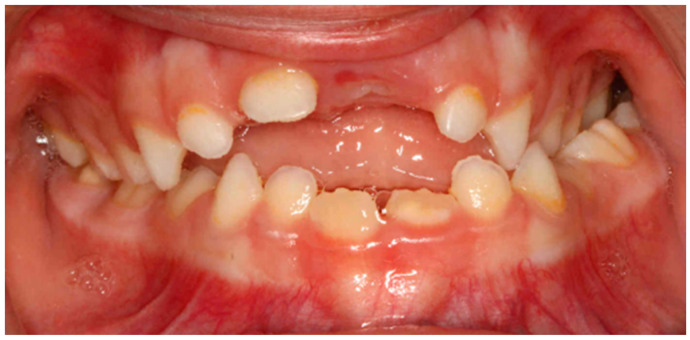
Intraoral photograph of an anterior open bite and unilateral posterior crossbite due to pacifier use for more than 2 years.

In the horizontal plane, a statistically significant relationship was found between oral respiration and unilateral posterior crossbite (*p* < 0.05) with a very strong relationship (V > 0.5) (Figure 3). Similarly, a unilateral posterior crossbite also had a statistically significant relationship with immature swallowing (*p* < 0.05) and with the persistence of the pacifier for more than 2 years (*p* < 0.05) (Table 4).

In the sagittal plane, no statistically significant relationship was found with any habit, although a greater relationship was observed between lip sucking and malocclusions in this plane (*p* = 0.059) (Table 5).

## 4. Discussion

The stomatognathic system is composed of static and dynamic structures and its harmonious functioning is based on the balanced relationship between them. Oral habits can affect the dentofacial growth of children, so the evaluation of the three planes allows us to understand their impact on the growth and development of children. In the present study, we have found statistically significant relationships between oral breathing, immature swallowing, pacifier sucking and lip sucking with the presence of different malocclusions, which we are going to analyse. 

In terms of prevalence, it should be noted that 72.64% of the children explored in our study had malocclusion in at least one plane of space. In the descriptive analytical study carried out by Festa et al. in 2021 [20] we can observe a notorious growth in the prevalence of malocclusions in recent years related to habits, where 81.4% had some alteration in occlusion. In the studies carried out by Paolantonio et al. [14] and Corrêa-Faria et al. [21] the prevalence of malocclusions was lower at 46% and 63.5%, respectively. This may lead us to think that over the years, malocclusions have increased due to the persistence of childhood habits.

Regarding oral breathing, different investigations have shown that it affects dentoskeletal development and masticatory function in children [22]. When breathing is performed through the oral cavity, the jaws and the muscles are affected. The characteristics of the clinical picture vary depending on the part of the airway that is altered, the health and biotype of the patient, as well as the time in which this habit acts [23]. We have found in this research that the malocclusion most related to this habit was posterior crossbite with a statistically significant relationship (*p* < 0.05) and a very strong relationship (V > 0.5). Although the relationship was not statistically significant, almost 50% of the children explored presented some malocclusion in the vertical plane with open bites being more frequent, coinciding with Daimon et al. [24]. However, other studies [20,25] observed a greater predisposition to Class II, since oral breathing caused by nasopharyngeal obstruction has been described to cause the clockwise rotation of the mandibular, often resulting in Class II malocclusion and a large protrusion.

Another oral habit that affects the dentoskeletal morphology is caused by alterations in the movements of the tongue, lips, and jaw during the act of swallowing, which causes what is known as atypical swallowing [5]. According to the American Academy of Pediatric Dentistry, this tongue thrust and force causes an open bite and a protrusion of the upper incisors, which we have observed in our results by finding the presence of open bites relative to the presence of this habit with a statistically significant relationship (*p* < 0.05). However, in this study, a statistically significant relationship with unilateral posterior cross-bites was also obtained. A different contribution is also made by the study of Rodriguez-Olivos et al. [26] in 2022, who found that 91.7% of the children with atypical swallowing presented an edge-to-edge bite.

It should be noted that non-nutritive sucking habits are a physiological activity during the first months of a child’s life, but if they are maintained they can affect the development of dental occlusion, including pacifier sucking. In the study by Caruso et al. [27], 79.79% of the children explored used the pacifier in the first three months of life, of which 43.49% prolonged its use for more than 2 years. Similar is the case with our study, where 72.64% of the children had also used the pacifier at some point in their childhood. According to the American Academy of Pediatric Dentistry, pacifier use is able to decrease the likelihood of digital sucking, as they already satisfy their need for sucking. In our study this could make sense, since the only children who presented thumb sucking had never resorted to pacifier use. In the literature, it has been found that children who presented this habit were more likely to have an anterior open bite than those who did not develop this habit [28]. The study by Schmid et al. [29] concluded that prolonged pacifier use (approximately 3 years) can lead to an open bite and posterior crossbite. Rai et al. [30] found among children in Nepal a significant association with crossbite with *p* = 0.01. However, we have not found a statistically significant relationship in the vertical plane but we agree in the horizontal plane between pacifier use for more than 2 years and the appearance of posterior crossbite, both unilaterally and bilaterally. Moreover, this coincides with the higher probability of having a posterior crossbite (2.39 times more) described by Belitz et al. [28] in 2022. In the sagittal plane, in 2020, Traebert et al. [31] pointed out that the prolonged use of pacifiers favours the appearance of Class II, although in our study there is no evidence of this.

Digital sucking is one of the first reflexes that very young babies can have, giving them a sense of security [32]. Children with a history of digital sucking are more likely to develop malocclusion, and there is evidence that the longer the duration of this habit, the more severe the development of malocclusion tends to be [33,34,35]. In the recent systematic review by Doğramacı and Rossi-Fedele [36], when comparing pacifier sucking with digital sucking, they observed that in children with digital sucking, the protrusion was even greater and that in mixed dentition an increase in the posterior crossbite and anterior open bite was observed. However, in the present study no such relationship was observed, probably because our sample of children with this habit was too small to be able to compare with the findings in the scientific literature.

Although the relationship was not statistically significant (*p* = 0.05), the habit of lip sucking is another of those highlighted in this study, as we observed a large number of patients with Class II who presented it. The American Academy of Pediatric Dentistry does relate this habit with a greater tendency to Class II. These patients place the lower lip behind the upper teeth, both at rest and when swallowing. The lower lip, interposed between the upper and lower incisors, ends up with the passage of time tilting the upper incisors out of the mouth and crowding the lower incisors inward. Another contribution was made by Lin et al. [37] in a recent study in 2023 that showed that children who had lip-sucking habits developed a higher prevalence of deep overbite than those who did not.

Regarding nutritional sucking, 81.1% of the children were breastfed and 61.3% were artificially breastfed, with no relationship found with malocclusion in any of the three planes. Consistent with the results of the current literature, breastfeeding is a positive factor in reducing the incidence of a posterior crossbite, and Class II skeletal, skeletal, and distoclusion in primary and mixed dentition [38,39]. Breastfeeding could be said to have a preventive effect on malocclusions by promoting proper growth and bone and muscle development [38,40,41]. It reinforces physiological nasal breathing [42,43] and is positively associated with the development of dental arches in the primary dentition in the transverse and sagittal planes [41,44]. Breastfeeding for a period equal to or greater than 6 months has been documented to act as a prevention factor for the acquisition of harmful oral habits. Other authors recommend up to 12 months of breastfeeding [15,45,46,47,48]. However, artificial feeding, used as an alternative method to breastfeeding, predisposes to the development of a malocclusion, according to the scientific literature. Different studies [49,50] have observed that artificial feeding was a risk factor for the development of malocclusions as it is associated with non-nutritive sucking habits and low muscle activity [51].

This study has some important limits. It should be noted that our data pertain to children sampled from a school in Seville. Another significant limitation of this study is the lack of a sample size calculation. In addition, in this study the sample size has been too small to establish firm conclusions. Therefore, studies with larger samples with a sample size calculation and different populations would be necessary that would allow more variables that can influence malocclusions to be correlated. Another limitation is the statistical approach chosen for a small size sample, so we must be cautious with the findings obtained in the statistical analysis. Furthermore, the types of malocclusion were only analysed from the dental point of view through a clinical examination and were not objectified by a cephalometric radiographic study, making it difficult to determine the extent to which an observed malocclusion is exclusively attributable to oral habits.

Due to the evident correlation between oral habits and malocclusions, and the greater possible persistence of habits currently, further research is recommended in this field. Likewise, it would be interesting to develop preventive programmes by educating and informing paediatricians, teachers, parents, etc., of the consequences at the dentoskeletal level that these habits cause. A diverse variety of approaches has been used to help children with stopping. These include advice, the removal of the comforting object, the application of an aversive taste to the digit or behaviour modification techniques, etc. Some of these interventions are easier to apply than others and less disturbing for the child and their parent; some are more applicable to a particular type of habit.

These oral habits related to malocclusions have repercussions on bone, dental, and muscle levels. Therefore, it is important to know what each of these habits consists of and to carry out early detection as soon as possible to prevent problems derived from them. The sooner they are removed, the less repercussions. Furthermore, early therapeutic management of these habits with myofunctional therapy and orthodontic appliances is one of the reasons why it is important to go to the paediatric dentist and orthodontist when the child is small. In addition, collaboration is needed between different specialists (paediatricians, allergists, speech therapists).

## 5. Conclusions

The prevalence and prolongation of habits in childhood is increasing, resulting in an increase in the predisposition to malocclusions in recent years. Despite the limitations of the present study, oral breathing could be related to vertical and horizontal malocclusions, with an open bite and posterior crossbite being the most common. Atypical swallowing would be capable of causing malocclusions, with an open bite being the most common. The prolonged use of a pacifier for more than 2 years would affect the appearance of malocclusions, mainly the open bite and posterior crossbite. Lip sucking habits seem to favour the tendency to Class II. The early detection of harmful habits at an early age is essential to prevent the establishment of malocclusions and thus promote the correct craniofacial development of the child.

## Figures and Tables

**Table 1:** Inclusion and exclusion criteria.

INCLUSION CRITERIA	EXCLUSION CRITERIA
5–12 years old	Children with craniofacial disorders
Signed authorisation	Orthodontic treatment
Adequate intraoral and extraoral examination	Incomplete erroneous survey

**Table 2:** Variables used for the study.

Study Variables	Sample (*n* = 106)	Study Variables	Sample (*n* = 106)	Study Variables	Sample (*n* = 106)
Gender Male Female	56 (52.8%) 50 (47.2%)	Nutritive suction -Maternal ○ No ○ ≤6 months ○ >6 months -Artificial ○ No ○ ≤2 years ○ >2 years	20 (18.9%) 31 (29.2%) 55 (51.9%) 41 (38.7%) 41 (38.7%) 24 (22.6%)	Malocclusion (vertical) No Open bite Overbite Edge to edge	63 (59.4%) 13 (12.3%) 24 (22.6%) 6 (5.7%)
Age 3–4 years 5–6 years 7–8 years 9–10 years 11–12 years	3 (2.8%) 34 (32.1%) 34 (32.1%) 26 (24.5%) 9 (8.5%)	Non nutritive suction Pacifier -No -≤2 years ->2 years Digital suction -No -Thumb suction Labial suction -No -Upper -Mixed	29 (27.4%) 56 (52.8%) 21 (19.8%) 104 (98.1%) 2 (1.9%) 99 (93.4%) 5 (4.7%) 2 (1.9%)	Malocclusion (horizontal) No Posterior crossed unilateral Posterior crossed bilateral Anterior crossed Scissors bite	93 (87.7%) 12 (11.3%) 1 (0.9%) 0 (0.0%) 0 (0.0%)
Dentition Temporal Mixed Permanent	21 (19.8%) 74 (69.8%) 11 (10.4%)	Overjet -No -<3 mm -≥3 mm	98 (92.5%) 3 (2.8%) 5 (4.7%)	Malocclusion (sagital) Class I Class II Class III	71 (67%) 14 (13.2%) 21 (19.8%)

**Table 3:** Association in the vertical plane.

	X SQUARE	V CRAMER
Oral Breathing	0.065	0.261
Immature deglution	0.011 *	0.324 **
Breastfeeding	0.879	0.107
Artificial breastfeeding	0.303	0.184
Pacifier	0.424	0.168
Digital suction	0.396	0.168
Labial suction	0.001 *	0.319 **

(*) *p* < 0.05, (**) V > 0.3.

**Table 4:** Association in the horizontal plane.

	X SQUARE	V CRAMER
Oral Breathing	0.000 *	0.504 ***
Immature deglution	0.027 *	0.261
Breastfeeding	0.621	0.111
Artificial breastfeeding	0.342	0.146
Pacifier	0.007 *	0.259
Digital suction	0.867	0.052
Labial suction	0.951	0.058

(*) *p* < 0.05 and (***) V > 0.5.

**Table 5:** Association in the sagittal plane.

	X SQUARE	V CRAMER
Oral Breathing	0.566	0.104
Immature deglution	0.714	0.080
Breastfeeding	0.849	0.080
Artificial breastfeeding	0.745	0.096
Pacifier	0.641	0.109
Digital suction	0.605	0.097
Labial suction	0.059	0.207

## Data Availability

Data will be available by asking the corresponding authors.
